# Natural Killer Cell Interactions With Myeloid Derived Suppressor Cells in the Tumor Microenvironment and Implications for Cancer Immunotherapy

**DOI:** 10.3389/fimmu.2021.633205

**Published:** 2021-05-05

**Authors:** Cristina Zalfa, Silke Paust

**Affiliations:** Department of Immunology and Microbiology, The Scripps Research Institute, La Jolla, CA, United States

**Keywords:** natural killer cells, myeloid derived suppressor cells, tumor mircroenvironment, natural killer cell immunotherapy, cancer

## Abstract

The tumor microenvironment (TME) is a complex and heterogeneous environment composed of cancer cells, tumor stroma, a mixture of tissue-resident and infiltrating immune cells, secreted factors, and extracellular matrix proteins. Natural killer (NK) cells play a vital role in fighting tumors, but chronic stimulation and immunosuppression in the TME lead to NK cell exhaustion and limited antitumor functions. Myeloid-derived suppressor cells (MDSCs) are a heterogeneous group of myeloid cells with potent immunosuppressive activity that gradually accumulate in tumor tissues. MDSCs interact with innate and adaptive immune cells and play a crucial role in negatively regulating the immune response to tumors. This review discusses MDSC-mediated NK cell regulation within the TME, focusing on critical cellular and molecular interactions. We review current strategies that target MDSC-mediated immunosuppression to enhance NK cell cytotoxic antitumor activity. We also speculate on how NK cell-based antitumor immunotherapy could be improved.

## Introduction

Tumorigenesis is a complex and dynamic process involving three stages: initiation, progression, and metastasis (1). Besides blood and lymphatic vessels, composed in part of vascular endothelial cells, the major constituents of the tumor microenvironment (TME) are a heterogeneous population of cancer cells, fibroblasts, immune and inflammatory infiltrated cells, and secreted protein elements of the extracellular matrix (ECM) (2). The functional and physical interactions of these tumor elements determine clinical outcomes. At the beginning of the nineteenth century, Virchow described cancer as originating from chronic unresolved inflammation (3). Many studies have demonstrated that cancer-associated inflammation plays a critical role in tumor formation, contributing to genomic instability and epigenetic modification, and regulating the creation of a protected TME to promote cancer proliferation and metastasis (4–6). Tumor-associated inflammatory cells are observed in human cancers from the earliest phases of carcinogenesis (7). The first line of defense, represented by natural killer (NK) cells and cytotoxic CD8^+^ T cells, usually recognizes and kills malignant cells; however, a few immunogenic cancer cell variants can escape immune recognition (8).

The TME orchestrates multiple mechanisms to impair the antitumor functions of immune cells. These mechanisms include destabilizing the innate cell compartment, composed of NK cells, macrophages, neutrophils, and dendritic cells (DCs), and suppressing adaptive immune cell (T and B)-mediated antitumor activity. The presence and recruitment of atypical innate and adaptative immune cells in the tumor site are thought to occur during both the early and later stages of tumor development (6, 9). Tumor immune surveillance also fails because immunosuppression and its associated chronic inflammation further destabilize tumor-fighting immune cells, defending rather than eradicating tumors (10). The TME supports the growth of cancer-associated fibroblasts, stromal cells, and endothelial cells, contributing to tumor-associated capillary and lymphatic vessels that support tumor growth (11). These mechanisms select potentially aggressive tumor clones early during tumor development (9).

This review will discuss the crosstalk between NK cells, myeloid-derived suppressor cells (MDSCs), regulatory T cells (Tregs), and the critical cellular and molecular mechanisms within the TME that impact tumor development, progression, and angiogenesis, as well as how current therapies target these immunosuppressive cells in the TME. We will also review different NK cell exhaustion mechanisms and novel approaches for enhancing NK cell therapeutic potential against tumors.

## Cytotoxic T Lymphocytes and NK Cells in Cancer

One of the adaptive and innate immune systems' essential activities is to kill infected and tumor cells. Mounting epidemiological and experimental evidence points to a critical role for cytotoxic T lymphocyte (CTL) and NK cell-mediated effector functions in host resistance to cancer. The collaboration between innate and adaptative effector cells can lead to tumor rejection (12, 13). CTL and NK cell activity has been linked to tumor immune surveillance and protection from cancer. Both cell types can form cytotoxic immunological synapses (14), which are specialized antigen-specific cell–cell junctions with a synaptic cleft to directly communicate and transduce highly controlled secretory signals between immune cells and their target cells (14). This mechanism, also present in NK cells, improves the efficiency of cytotoxic cell-mediated killing (15). Higher numbers of tumor-infiltrating CTL and/or NK cells are a favorable prognostic indicator for many cancer types (16, 17). T cell activation occurs when a clonal T cell receptor (TCR) is triggered by a tumor-derived antigen presented on class I human leukocyte antigen (HLA-I), in combination with co-receptor ligation and co-stimulation, leading to CTL activation, proliferation, cytokine and chemokine secretion, and tumor cell killing (18). Similar to CTL cells, NK cells are also cytotoxic lymphocytes and important tumor fighters.

NK cells do not express a TCR but instead have many activating receptors and also inhibitory receptors, which bind major histocompatibility complex class I (MHC-I) (19, 20). MHC-I is often downregulated by infected and malignant cells to avoid CTL killing. NK cells sense this “lack of inhibition” and are further activated by tumor cell-expressed stress ligands that ligate NK cell-expressed activating receptors (20–22). NK cells secrete cytokines, chemokines, pore-forming proteins (perforin), and cytotoxic mediators (granzymes) that trigger target cell apoptosis upon activation. A potent NK cell-activating receptor is NK group 2 member D (NKG2D), and NKG2D ligands are commonly upregulated on tumors (23).

Substantial evidence supports the conclusion that NK cells play a crucial role in eliminating tumors and tumor metastases (24). First, low cytotoxicity in peripheral blood (PB) NK cells correlates with a higher risk of developing cancer (25). Second, in many types of cancer, NK cells exhibit an altered phenotype and hypo-functionality (26, 27). Third, in mice, resistance to NK cell killing favors polyclonal metastasis (28). Several mechanisms contribute to NK cell exhaustion, including modulating adhesion and epithelial genes, decreasing the expression of NK cell-activating ligands (28), and the suppressive effects of regulatory immune cells and soluble factors within the TME (29).

## NK Origin, Development, and Tissue Distribution

NK cells are bone marrow (BM)-derived granular lymphocytes that lyse target cells rapidly and continuously upon activation (30). In the BM, NK cells develop from CD34^+^ hematopoietic stem cells through a common lymphoid progenitor (CLP) intermediate that can seed and develop further in lymphoid and non-lymphoid organs (31). NK cell maturation requires several cytokines, among which interleukin (IL)-15, released by BM stromal cells, is crucial for the differentiation of CLPs toward the NK cell lineage (32). NK cells are well-represented in the PB, spleen, and BM and are found in most organs, including the liver, lungs, skin, gut, lymph nodes, tonsils, uterus, thymus, kidney, pancreas, and adipose tissue. (33, 34). Their recruitment to different tissues depends on the expression of several chemokine receptors and is reviewed elsewhere (35, 36).

Human NK cells express the hematopoietic cell marker CD45, the glycoprotein CD56 and mature NK cells, and the cluster of differentiation molecule CD16 also known as Fc receptor FcγRIIIa, but they do not express any T or B cell receptors. NK cell maturation is generally assessed by the amount of NK cell-expressed CD56 and CD16 expression. Specifically, CD56^dim^ and CD56^bright^ subsets show profound differences in cytokine secretion, response to cytokines, and killing efficiency. CD56^dim^ CD16^bright^ NK cells in the PB are about 90% of the total NK cell population and include the alloreactive NK cells described as “mature” with a higher cytotoxic potential. The remaining 10% of NK cells are CD56^bright^ CD16^dim^ and reside in the lymphoid tissue; they are considered “immature or unlicensed.” These NK cells are more sensitive to cytokine stimulation, which will activate the secretion of a variety of cytokines, including interferon-gamma (IFN-γ), tumor necrosis factor-alpha (TNF-α), IL-5, IL-10, and IL-13 (37–39).

A sophisticated array of germline-encoded activating and inhibiting receptors regulates NK cell development and, subsequently, their activation (40). Upon activation, NK cells employ several mechanisms of target cell killing and secrete chemokines and cytokines to interact with other immune cells (41, 42). NK cells are traditionally defined as innate immune response cells because they lack recombinase-dependent clonal antigen receptors (RAG) (43). Nonetheless, recent findings have revealed that NK cells display adaptive immunity features (44–47), including several developmental and functional characteristics of the adaptive immune system (43, 48–51). These similarities include vaccination or sensitization-dependent antigen-specific immunological memory (45, 46, 52–55). How NK memory impacts the tumor-specific NK cell response is currently unknown.

## Antitumor Responses of NK Cells

Many studies have demonstrated that NK cells can kill tumor cells (56). NK cells survey their environment with a distinct receptor repertoire, including activating and inhibitory receptors, adhesion molecules, and cytokine and chemokine receptors (57). NK cells recognize the expression of HLA-I, also called MHC-I, on autologous cells. This interaction is generally inhibitory and prevents NK cells from attacking healthy host tissue. Specifically, HLA-I or MHC-I binds NK cell inhibitory receptors, including killer cell immunoglobulin-like receptors (KIRs) in humans, Ly49 in mice, and CD94/NKG2A (58, 59). In contrast to infected or malignant cells, healthy nucleated cells express robust levels of HLA-I/MHC-I molecules and escape NK cell immune attack. However, during malignant transformation or viral infection, the expression of MHC-I antigens on the cell surface can be downmodulated. This variation in the expression of MHC-I molecules on target cells (missing self) reduces the strength of inhibitory signals delivered to NK cells, thus promoting NK cell activation. NK cells survey tissues for low MHC-I molecule expression (60, 61) and for the expression of activating ligands, such as the NKG2D ligands (62, 63), and ligands for the natural cytotoxicity receptors NKp30 and NKp44 in humans and NKp46 in humans and mice (64).

NK cells also have the unique ability to exert antibody-dependent cell-mediated cytotoxicity (ADCC) upon engagement of CD16 with the Fc portion of the antibodies (65). Activation through these receptors elicits rapid target cell killing by several mechanisms. NK cells form an immunological synapse with target cells and kill them rapidly by secretion of lytic granules that contain an arsenal of effector molecules (perforin, granzymes) and cytokines (IFN-γ, TNF-α) that induce cell death in targeted cells (66–68). Perforin and granzyme are proteins that play a significant role in cell-mediated cytotoxicity. These molecules are expressed in NK cells, and several cytokines regulate their level of expression in CTLs. The role of perforin, which is involved in T cell- and NK cell-mediated target cell lysis, was demonstrated in mice lacking perforin with respect to their capability to eradicate a syngeneic lymphoid tumor mammary adenocarcinoma (69). Smyth and coworkers demonstrated that mice with lymphoma and deficient in the pore-forming protein perforin [(pfp)-deficient] showed an increased number of premalignant cells than their immunocompetent counterparts. In fact, pfp-deficient mice were 1,000-fold more susceptible to tumor (70), demonstrating that lymphocyte-mediated cytotoxicity plays an essential role in promoting host resistance to spontaneous tumor formation. NK cells also express several TNF superfamily proteins and death-inducing ligands, such as TNF-related apoptosis-inducing ligand (TRAIL) and FAS ligand (FASL), which induce target cell apoptosis via binding to their corresponding receptors (TRAIL-R and FAS). Activated NK cells also produce growth factors, such as granulocyte-macrophage colony-stimulating factor (GM-CSF) and chemokines (XCL1, CCL3, CCL4, and CCL5) l (26, 33, 71–73).

## NK Cells in Solid Tumors

Genetic alterations in oncogenic pathways associated with an aberrant inflammatory milieu (74, 75), abnormal activations of transcription factors (nuclear factor kappa-light-chain-enhancer of activated B cells [NF-κB] and signal transducer and activator of transcription-3 [STAT3]) (76, 77), and hypoxia (78) may contribute to development and maintenance of the TME. The TME is responsible for tumor onset and progression by orchestrating cell growth, proliferation, malignancy, and immune escape processes. TME can impair and “polarize” the innate, adaptive, stromal, and endothelial cell compartments by several mechanisms (2, 79–82). Tumor cells, MDSCs, and tumor-associated cells, such as tumor-associated fibroblasts (TAFs) and endothelial cells (83), can contribute to tissue modifications of the ECM by matrix metalloproteases (MMPs) and fibroblast activation proteins and the release of soluble factors (basic fibroblast growth factor [FGF], platelet-derived growth factor, hepatocyte growth factor, insulin-like growth factor), chemokines (CCL2, SDF1a/CXCL12, CXCL8), and immunosuppressive cytokines, such as transforming growth factor beta (TGF-β), IL-10, and IL-6 (84, 85). Due to the adverse TME, immune cells lose their ability to reach, recognize, and target tumor cells (2, 79, 86, 87). The TME impairs immune cell homing to the lymphoid organs promoting tumor immune cell escape, invasiveness, and angiogenesis (88–90). Thus, chronic inflammation orchestrated by immunosuppressive mediators of the TME supports tumor progression by exhausting immune cells, such as NK cells (74, 78).

NK cells also express inhibitory receptors targeting non-MHC molecules on healthy cells. One of these inhibitory receptors expressed on NK cells is killer cell lectin-like receptor G1 (KLRG1), a well-conserved member of the C-type lectin receptor superfamily. KLRG1 is known for its role in NK cell maturation, development, and homeostasis (91). Recently, a new role for KLRG1 has emerged as an inhibitory receptor impacting NK cell function in tumor surveillance. Impairment in the migration and/or retention of NK cells in the BM has been observed in multiple myeloma (MM). BM localization of the more functional NK KLRG1^−^ subtypes is impaired in MM by altering the chemokine microenvironment (increasing chemokine [CXC] ligand 9 [CXCL9] and CXCL10 and reducing CXCL12 expression in BM) in a mouse tumor model of an early cancer growth stage (88). This is due to significant dysregulation of the CXCR3 and CXCR4 chemokine receptor/ligand axes, influencing NK cell responses (88, 92). Using murine models of chronic NK cell stimulation, Alvarez and colleagues have identified a “phenotypic signature of NK cell exhaustion,” characterized by upregulation of KLRG1 and downregulation of the activating receptor NKG2D (93). KLRG1 ligands, such as E- and N-cadherin, were upregulated in tumor specimens from patients with melanoma, breast, prostate, and colorectal cancer (94). The same authors showed that anti-KLRG1 antibody monotherapy in a 4T1 breast cancer mouse model enhanced tumor control compared to controls (94). Tata and colleagues also demonstrated that KLRG1-deficient mice had significantly fewer lung tissue tumors than wild-type controls (95).

Several studies have demonstrated the prognostic significance of tumor-infiltrating lymphocytes and their antitumor actions in cancer (16, 17, 96–99). It has recently become clear that CTL and NK cell cooperation are essential in many types of tumors (100–110). Using mouse models of mastocytoma (mice heterozygous for the H-2Ld/P1A35–43-specific TCR transgene: TCRP1A on the DBA/2, B10.D2; TCRP1A tg B10.D2[×DBA/2] F1; RAG-1°/°B10.D2), a study demonstrated that the frequency of cancer antigen-specific T cell precursors and the rate of antigen variants can contribute to the efficacy of adaptive T cell responses to cancer (107). Moreover, the efficiency of an effective antitumor antigen-specific T-cell response can depend on the complementary interaction between effector T cells and NK cells (107). Another study also demonstrated that the NK cell's antitumor effect requires interaction with specific activated tumor antigen-CD8^+^ T cells (12). However, further studies are necessary to clarify the mechanism of interaction between NK cells and specific effector T cells.

In clinical trials studying solid tumors, impaired NK cell function correlated with a poor prognosis in patients with advanced disease (111, 112). The TME plays a critical role in reducing NK cell persistence and trafficking in the tumor site by inhibiting NK cell activation, leading to tumor invasion and metastasis (26). Moreover, several studies showed anergic and hypo-functional NK cells (26, 27, 113–120), including tumor-associated NK cells in the PB and tumor-infiltrating NK cells within the tumor tissue. These CD56^bright^ CD16^low/−^ Perforin^low^ NK cells even exhibited pro-tumorigenic functions and pro-angiogenic activities (80, 121–124) and have been identified preferentially in many solid tumors (80, 87, 115–121, 123, 125–129). Some of these NK cells also downregulated their expression of NKG2D, impairing their antitumor functions further.

## MDSCs and Tregs in Cancer

### Development and Phenotypes of MDSCs

**Figure 1:**
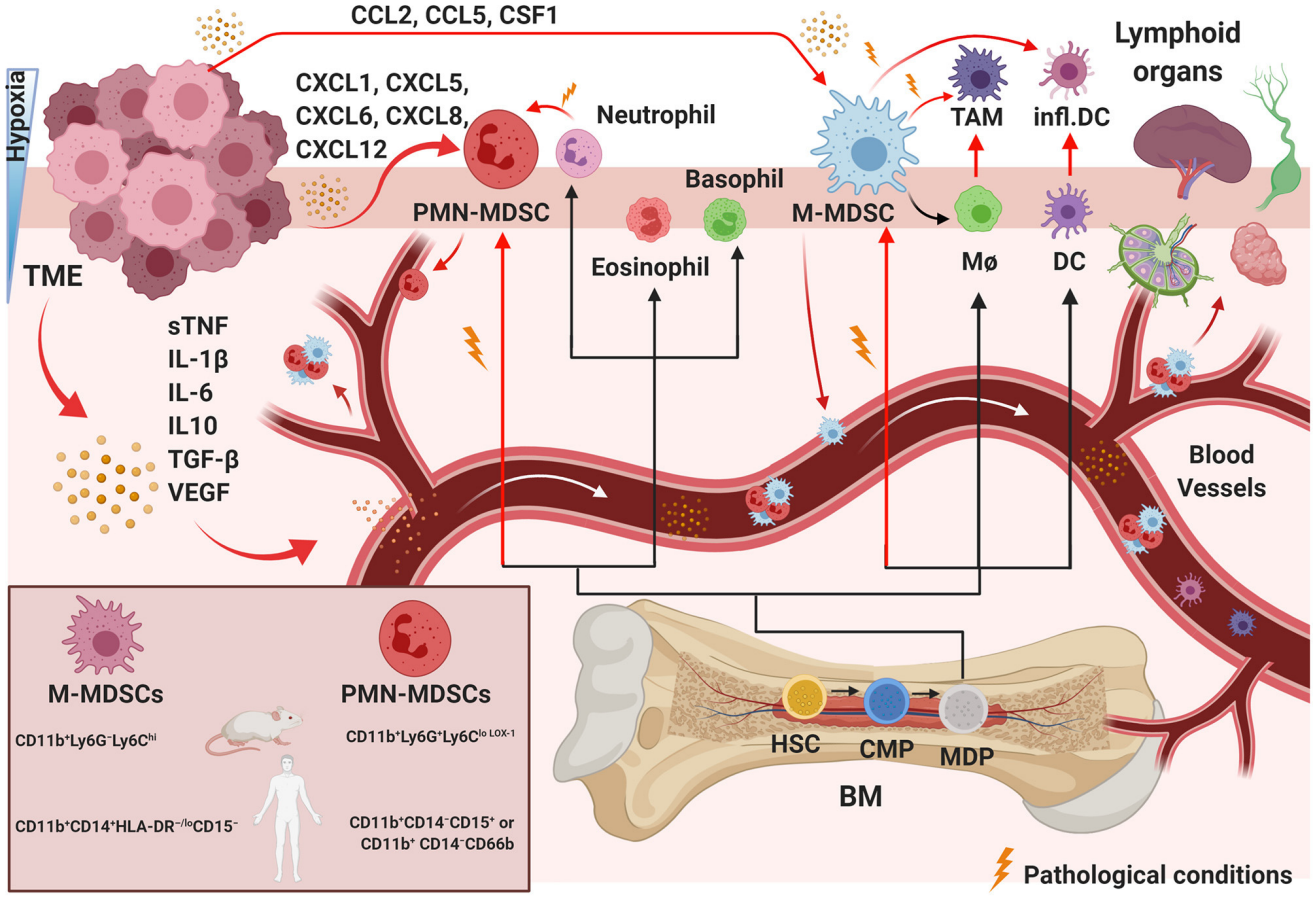
Pathological myelopoiesis in cancer. Under physiological conditions and chronic inflammation, hematopoietic progenitor cells (HPCs) differentiate via common myeloid progenitor cells (CMPs) into monocytic/dendritic progenitor cells (MDPs). Myelopoiesis is altered under pathological conditions, such as in a tumor. Tumor-immunosuppressive factors produced in the TME alter myelopoiesis, leading to aberrant differentiation and accumulation of myeloid lineage cells. The black lines show the normal pathways of myeloid cell differentiation from immature myeloid precursor cells to dendritic cells (DCs), macrophages (MΦ), and granulocytes, as depicted. The red lines indicate the aberrant pathways of myeloid cell differentiation that arise in cancer, in which the TME promotes the development of immunosuppressive populations, including monocytic myeloid-derived suppressor cells (M-MDSCs), polymorphonuclear myeloid-derived suppressor cells (PMN-MDSCs), inflammatory DCs, and tumor-associated macrophages (TAMs). The MDSC mouse and human phenotypes are shown in the left square on the bottom. BM, bone marrow; sTNF, soluble tumor necrosis factors. Created with BioRender.com.

Common myeloid progenitors differentiate from hematopoietic stem cells in the BM. Later, they migrate to the peripheral lymphoid organs and differentiate into myeloid cells. This pathway involves granulocyte-macrophage progenitor and various myeloblast intermediate precursors, including common monocyte progenitors (130) (Figure 1). Immunological stress, as well as cancer, prolonged inflammation, trauma, and autoimmune disorders, can impair the differentiation of these immature myeloid cells (131). Tumor-associated myeloid cells are mainly represented by tumor-associated macrophages (TAMs) and MDSCs, which are one of the crucial players within the TME (Figure 1). The TME often subverts immunosurveillance by generating MDSCs with strong immunosuppressive activity and functional plasticity (130). MDSCs are a heterogeneous population of myeloid-derived cells represented by myeloid progenitors, immature granulocytes, DCs, and macrophages. Therefore, the characteristics that separate MDSCs from other myeloid cells are still under investigation. It is widely accepted that MDSCs are divided into two main subsets: granulocytic or polymorphonuclear (PMN)-MDSCs and monocytic (M)-MDSCs, cells showing a phenotype and morphology similar to neutrophils and macrophages, respectively (132–136) (Figure 1). However, MDSC subtypes can be distinguished from neutrophils and TAMs that are present in the TME (137, 138). Studies have shown additional mechanisms to describe the evolution and roles of these polarized neutrophils in the TME, and some evidence supports the idea that these cells are similar to MDSCs and could be described as PMN-MDSCs (139–141), which represent the most prevalent cells in several types of tumors (142, 143). MDSCs with granulocyte and monocyte hallmarks have genomic profiles, biochemistry, and *in vitro* properties that differ from neutrophils, monocytes, and DCs (144). Recently, whole-transcriptomic and proteomic analyses (134, 145–147) provided specific gene expression patterns for the characterization of these different cell types. Cell-surface markers have been identified to distinguish MDSC-specific phenotypes from TAMs, tumor-associated neutrophils, and neutrophils. M-MDSCs can be separated from TAMs by their differential expression of F4/80, M-CSF and CD115^high^, Ly6C^lowtointermediate^, IRF8^low^, and S100A9^verylow^ (148). In contrast, PMN-MDSCs are CD11b^+^Ly6G^+^Ly6C^loLOX−1^ in mice and CD11b^+^CD14^−^CD15^+^ or CD11b^+^ CD14^−^CD66b in humans, while M-MDSCs are CD11b^+^Ly6G^−^Ly6C^hi^ in mice and CD11b^+^CD14^+^HLA-DR^−/*lo*^CD15^−^ in humans (Figure 1). A low percentage of MDCSs (around 3%) consists of a mixture of more immature progenitors and precursors with myeloid-colony-forming activity termed “early-stage MDSCs” (e-MDSC) and are described as Lin^−^HLA-DR^−^CD33^+^ (132, 144). These and other novel marker combinations are currently under further investigation (133, 134, 149).

The number of MDSCs is associated with the clinical cancer stage and metastatic disease (150). Thus, MDSCs could be a potential biomarker of disease in several types of cancer. For example, the frequencies of MDSCs change during tumor progression in glioma (151) and cervical cancer (152). The frequency of peripheral PMN-MDSCs has been correlated with cancer prognosis, while the percentage of M-MDSCs has been shown to be higher in patients with advanced cervical cancer (152). Moreover, PMN-MDSCs' rate is negatively correlated with CD8^+^ T cells' rate (151, 152). MDSCs have been reported as prognostic markers in non-small cell lung cancer (NSCLC) (153); breast and colorectal cancer (154); gastric, esophageal, and pancreatic cancer (155); and melanoma (156). Therefore, MDSCs can also be used as a predictive marker for immunotherapy. Additionally, a diminished number of MDSCs helped to eradicate metastatic disease after the removal of primary tumors in a mammary carcinoma model (157).

### Recruitment and Expansion of MDSCs in Tumors

While absent in normal physiological conditions (144), MDSCs can be detected in the BM, blood, spleen, tumor, and lymph nodes in pathological conditions (Figure 1). MDSCs have been shown to increase significantly in early- and late-stage cancer in preclinical animal models and human tumors (6, 145, 151). Upon their recruitment, MDSCs gradually expand in the TME and support the development of an immunosuppressive tumor environment by interacting with several components of the innate and adaptive immune systems (158, 159) and by stimulation of neo-angiogenesis (160, 161). Vetsika et al. described all phases of this process (162); here, we will summarize. The network of transcriptional regulators that directs MDSC development can be combined into two partially overlapping groups: (i) factors promoting myelopoiesis and avoiding differentiation of mature myeloid cells and (ii) factors contributing to pathologic activation of MDSCs. In different types of mouse and human cancers (132, 144, 163–166), MDSCs are gradually recruited and increase in the TME. They support tumor progression through non-immune activities by stimulating pre-metastatic niche formation, invasion (167, 168), and inducing pro-tumor angiogenesis (169). Some authors have proposed a “two-signal model” for describing how MDSCs can acquire the modifications guiding their pathological activation, immunosuppressive activity, and expansion in the TME under tumor pathologic signals (170, 171). Myeloid cells exposed to pathological conditions (autoimmunity, cancer, trauma, graft vs. host disease, and infections) can be activated in response to damage-associated molecular pattern molecules, pathogen-associated molecular pattern molecules, or pro-inflammatory cytokines (144). Because patient blood has been observed to have increased tumor-released macro- and micro-vehicles during tumor progression, tumor niches could potentially gather MDSCs from the BM by releasing exosomes. Their contents have been demonstrated to reprogram target cells in different types of cancer, increase the mobility of the progenitor myeloid population to the tumor site, and increase tumor immunosuppression (162).

MDSCs migrate in response to several chemo-attractant molecules released from cells in the TME using two main pathways: PMN-MDSC migration includes the secretion of CXCL1, CXCL5, CXCL6, CXCL8, and CXCL12, and M-MDSCs respond to CCL2, CCL5, and CSF1 (172) (Figure 1). The TME can guide the differentiation of incoming MDSCs in several different directions. The commitment of myeloid progenitor and precursor cells into MDSCs can be triggered by immunosuppressive cytokines released in the TME, such as soluble tumor necrosis factors (sTNF), IL-1β, IL-6, IL10, TGF-β, and vascular endothelial growth factor (VEGF) (173, 174) (Figure 1). MDSC phenotypes develop under conditions of acute or chronic inflammation, stress and hypoxia, high concentrations of oxidative molecules, and reduced nutrients (172, 175) (Figure 1). Hypoxia, specifically hypoxia-inducible factor 1 alpha (HIF-1α), appears to be one of the most critical stresses (35, 56) and was shown to be essential in M2-type TAM generation from Ly6C^hi^ monocytes inside a tumor (176). During hypoxia, immunomodulatory proteins and chemokines also mediate the differentiation of TAMs or M2 macrophages (177) from M-MDSCs or/and guide later events in tumor progression (177, 178).

Because M-MDSCs have a longer lifespan (179), their differentiation has been studied more extensively than that of PMN-MDSCs. TAMs can be derived from tissue-resident macrophages proliferating *in situ* in pancreatic and mammary tumors (180, 181). Several studies have shown the ability of M-MDSCs to differentiate into TAMs after migrating from the spleen to a tumor (178, 180, 182, 183), and TAMs can be “regenerated” by the arrival of new M-MDSCs from other organs during tumor progression (184–186) (Figure 1). MDSCs can also differentiate into DCs and fibrocytes during cancer progression (187). Moreover, Ly6C^hi^, Ly6CX3CR1, and Ly6C^+^CCR2^+^ monocytes can differentiate into TAM subsets (Figure 1) in mammary adenocarcinoma, lung adenocarcinoma, and lung carcinoma models (188, 189). In the chronic inflammation present in tumor tissues, IL-18 can support the function of TGF-β1 that is produced and activated by M2-polarized TAMs (190, 191). IL-18 promotes the differentiation of CD11b^−^ BM progenitor cells into M-MDSCs and increases their suppressive functions, including arginase expression (ARG1) and NO secretion (192). MDSCs induced by IL-18 can inhibit CD4^+^ T cell proliferation and IFN-γ production (192), contributing to the negative regulation of immune responses in tumors through immunosuppressive functions.

### Treg Origin and Development in the TME

CD4^+^CD25^+^ Tregs are a subpopulation of suppressor T cells that mediate immune homeostasis, maintain peripheral tolerance, and prevent immune and auto-immune disease by suppressing autoreactive T cells (193). These cells regulate immune responses in the context of immunity and infections differently (194, 195). In cancers, Tregs are linked to the development of an immunosuppressive TME, promoting immune evasion and cancer progression and preventing antitumor immunity (196–198). Tregs represent about 1–3% of CD4^+^ T cells in human tumors and about 10% in rodents. These cells express cell surface molecules associated with activated/memory T cells, CD25, FoxP3, CD45RBlow, CD62L, CD103, cytotoxic T-lymphocyte associated protein 4 (CTLA-4), and glucocorticoid-induced TNF receptor (199). An increase in Tregs prevalence has been shown in several tumor malignances (7), and they are recruited and expand within the TME via several mechanisms (200). We will summarize these stages in the following steps. Tregs are recruited into tumors in response to chemokines secreted by tumor cells and innate immune cells. Tregs then expand and proliferate in response to tumor-derived factors (TGF-β, adenosine, VEGF, and IL-10) within the TME. TGF-β and adenosine, released from cancer cells and also MDSCs, seem to play a key role in generating suppressive CD25^+^ FoxP3^+^ Tregs from non-suppressive CD25^−^ FoxP3^−^ conventional Tregs. The recruitment of Tregs occurs at early tumor stages, as demonstrated by their presence in pre-malignant lesions, and their prevalence increases with pancreatic and breast tumor progression and worsening clinical outcomes (114, 201–203). Moreover, it has been shown that the depletion of Tregs cells in pancreatic ductal adenocarcinoma slows tumor growth and prolongs survival (204–207).

### Crosstalk Between MDSCs and Tregs

**Figure 2:**
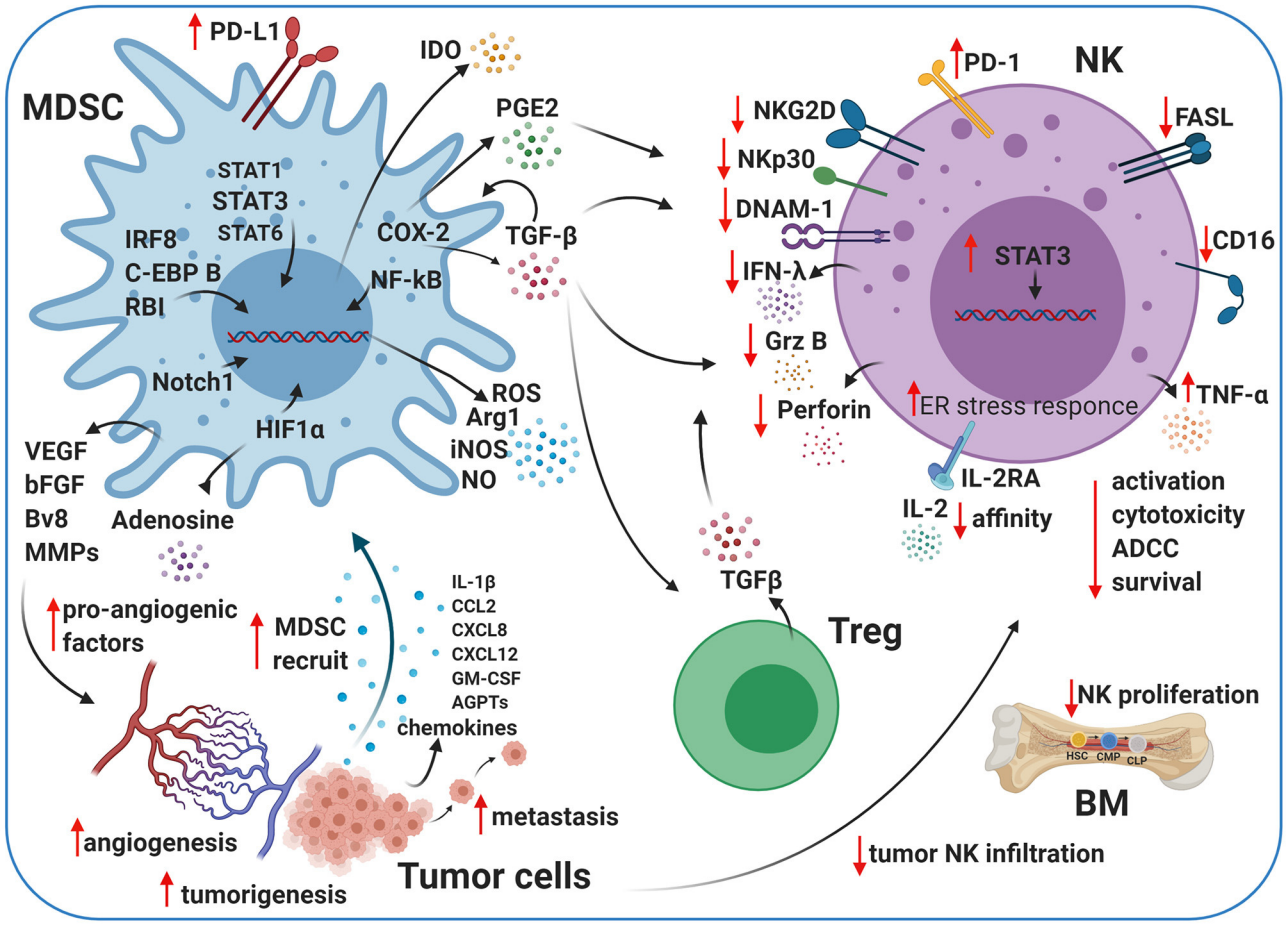
Crosstalk between MDSCs, NK cells, and Tregs in the TME. Myeloid-derived suppressor cells (MDSCs) and natural killer (NK) cells crosstalk within the TME. The cartoon shows the immunosuppressive activity of MDSCs and regulatory T cells (Tregs) on NK cells using different pathways. MDSCs impair NK cell activation, cytotoxicity, survival, and maturation by several secreted factors, including transforming growth factor beta (TGF-β), indoleamine 2,3-dioxygenase (IDO), nitrogen oxide (NO), reactive oxygen species (ROS), and prostaglandin-E2 (PGE2). MDSCs contribute to tumor angiogenesis by releasing pro-angiogenic factors, such as adenosine and vascular endothelial growth factor (VEGF), under pathological conditions. Created with BioRender.com.

MDSC expansion in PB is directly correlated with poor clinical outcomes (208–210). MDSCs can support the conversion of naive CD4^+^ T cells into Tregs by secreting retinoic acid and TGF-β (Figure 2), promoting the trans-differentiation of Th17 cells into Foxp3^+^ Tregs (211). Moreover, MDSCs can also induce Tregs immunosuppressive functions by mediating the release of IL-10 and IFN-γ (162). Tumor-infiltrating M-MDSCs express high levels of C-C chemokine receptor type 5 (CCR5) ligands and recruit high numbers of Tregs into the TME (212), establishing an additional cooperative network between MDSCs and Tregs (24, 35). Therefore, Tregs are accumulated in the TME and produce VEGF, which promotes angiogenesis (161, 213). By using light-sheet fluorescent microscopy, Siret and colleagues demonstrated direct interactions between MDSCs and Tregs in pancreatic ductal adenocarcinoma (214), and *in vivo* depletion of MDSCs significantly reduced the Tregs population in pancreatic tumors (215). Furthermore, video-microscopy and *ex vivo* functional assays have demonstrated that MDSCs can induce Treg cells by cell–cell-dependent contact at different stages of human cancer, and Tregs can also affect the survival and/or the proliferation of MDSCs (214). The molecular mechanisms guiding MDSC/Tregs interplay are not fully understood. The role of co-stimulatory molecules, protein membranes, and receptor candidates, respectively, such as PD-L1 (216), CD80 (217), and CD40 (218), is currently under investigation. Together, Tregs and MDSCs contribute to establishing an immunosuppressive TME in multiple solid neoplasms (114, 201, 202, 214).

## Cytokines and Other Mediators in MDSC-Mediated NK Cell Regulation

In addition to cell-intrinsic defects caused by chronic stimulation (219), an immunosuppressive TME represses CTL and NK killing (29, 220) via the recruitment of other cells types, such as MDSCs, M2 macrophages (221), and Tregs (222), which contribute to immune exhaustion via the expression of inhibitory ligands, suppressive cytokines, and tumor-promoting factors (221, 223). MDSCs display potent immunosuppressive activity and play a critical role in regulating tumors and metastasis development (144). MDSCs can impair CD8^+^ CTLs and NK cells directly by influencing the pro-tumor TME (224). Their contribution to regulating T lymphocytes is well-described, while their interactions with other immune cells, such as NK cells, DCs, or macrophages, in the TME are less understood (158). The suppressive effects of MDSCs are mediated through cell–cell contact, as well as the secretion of soluble factors, and result in antigen-specific or antigen-non-specific suppression of T-cell responses (225). The presence of MDSCs in tumors is associated with chronic inflammation and antigen-specific tolerance by T cells (226). MDSCs can also regulate the innate immune response by inhibiting NK cell functions (227–230) and/or by modulating macrophages' cytokine production (159, 225). The immunosuppressive activity of MDSCs plays an essential role in the regulation of the NK cell response to the tumor. *In vitro* co-culture of MDSCs and NK cells showed a reduction in NK cell-mediated cytotoxicity and higher tumor cell tolerance. MDSCs inhibit antitumor responses in part through immune checkpoint inhibition (ICI), including programmed death (PD)-1/PD-L1, galectin-9/T-cell immunoglobulin domain and mucin domain 3, and CTLA-4/B7 interactions (231).

STAT3 regulates NK cell biology at several levels, including activation, cytokine/cytolytic-mediated functions, and interactions with other immune system components (232). Many growth factors and cytokine receptors signal through STAT family transcription factors. STATs are impaired in several types of cancer and play a crucial role in innate and adaptive immunity (225, 233–235). In particular, STAT3 regulates several pathways involved in NK cell development, cytotoxic activities, and killing (232). Similarly, several cytokines, transcription factors/regulators, and signaling pathways are involved in the expansion and differentiation of MDSCs. These include interferon regulatory factor 8 (IRF8), CCAAT-enhancer-binding protein, and retinoblastoma protein; signaling pathways STAT3, STAT1, STAT6, Notch, NF-κB, and cyclooxygenase 2 (COX-2); and endoplasmic reticulum. Stress pathways are involved in MDSCs' expansion (170) (Figure 2) and include STAT3 activation in e-MDSC subtypes, which is critical for NF-κB activation and increasing indoleamine 2,3-dioxygenase (IDO), the release of which inhibits NK cell activation, proliferation, and effector functions (236). The molecular mechanisms regulating PMN-MDSC and M-MDSC populations differ from the afore-described pathways and are currently under investigation. Danvatirsen, a STAT3 antisense oligonucleotide, reversed the immunosuppressive TME and enhanced immune activity, as well as checkpoint blockades, in patient tumor samples from two phase I clinical trials and murine models (237).

Tumor-derived IL-1β release into the TME has been shown to enhance the recruitment of specific MDSCs during chronic inflammation (173). The augmented suppressive potential of IL-1β-induced MDSCs in mice was due to the activity of a novel subset of MDSCs lacking Ly6C expression (173). When these cells are prevalent under inflammatory conditions, they can impair NK cell development and function *in vitro* and *in vivo* by reducing the expression of the NK cell activating receptor NKG2D (173). Ly6C^−^ MDSCs may be a valuable therapeutic target.

Tumor-derived prostaglandin-E2 (PGE2) may also play a crucial pro-tumor role in inducing MDSCs, mainly via COX-2 (238) (Figure 2). Monocytes exposed to PGE2 acquire MDSC-like functions, gaining the ability to inhibit NK cells via TGF-β (239, 240). High levels of TGF-β in the plasma were observed in patients with advanced tumors and correlated with worse outcomes (241, 242). TGF-β is released from cancer cells and can increase the expansion of M-MDSCs (243), recruit suppressive cells to the TME (MDSCs, Tregs, DCs, and stromal cells), and compromise the function of NK cells (230, 244–246), cytotoxic CD8^+^ cells, DCs, Tregs, and macrophages (247). NK cells exposed to MDSCs secrete less IFN-γ and downregulate NKG2D and CD247 *in vitro* and *in vivo* (229, 230, 248, 249). TGF-β not only impairs NK cell functions by downregulating the expression of activating receptors (NKG2D and NKp30) and inhibiting their transcription but also downregulates tumor cell-expressed NKG2D ligands (250). Thereby, NK cells lose their capacity to recognize and kill tumor cells via NKG2D (250). Furthermore, TGF-β inhibits CD16-mediated IFN-γ production and ADCC in human NK cells through mothers against decapentaplegic homolog 3 (SMAD3) activation (251) and affects CD34^+^ hematopoietic progenitors by inhibiting the maturation of CD56^bright^CD16^+^ NK cells fraction in the PB (246). The incubation of PB-NK cells with stromal cells, isolated from decidual tissue conditioned media, mimicked the suppressive effects of TGF-β1. NK cell interactions with CXC chemokine ligands and progesterone at the maternal–fetal interface after TGF-β1 exposure resulted in the reduction of CD56^bright^CD16^+^ NK cells and induced decidual-like NK cells that showed an exhausted phenotype (246). As such, STAT3 blockade and TGF-β inhibition improve tumor immune surveillance by NK cells (252). Specifically, tumor-infiltrating and tumor-associated NK cells from STAT3-deficient tumor-bearing mice express enhanced levels of NKG2D, CD69, FASL, granzyme B, perforin, and IFN-γ, reducing tumor growth and improving survival (252, 253). TGF-β signaling is deregulated in many diseases, including cancer. In early-stage tumor cells, this pathway has tumor-suppressor functions, including cell-cycle arrest and apoptosis (254). TGF-β signaling in late-stage cancer can promote tumorigenesis, angiogenesis, metastasis, and immunosuppression (254–256). MDSCs release TGF-β in the TME (144, 163, 175), and NK cell anergy (Figure 2) correlates with the marked increase of MDSCs in the liver and spleen in orthotropic liver cancer-bearing mice (230). Also, MDSCs prevent cytotoxicity, NKG2D expression, and IFN-γ production by NK cells (Figure 2) *in vitro* and *in vivo* through membrane-bound TGF-β (230).

IDO is an intracellular enzyme, and it regulates tryptophan catabolism into kynurenine (257, 258), which inhibits the proliferation and function of NK and T cells (259–261). IDO synthesized by MDSCs impairs NK cell activation, development, and expansion, resulting in dramatically decreased expression of NKG2D and DNAM-1 and limiting IFN-γ secretion (262, 263) (Figure 2).

ARG1 and reactive oxygen species (ROS), soluble factors secreted by MDSCs, also impair NK cell functions (Figure 2) in cancer models *in vivo* (248, 264). Moreover, several pro-inflammatory cytokines have been identified to mediate MDSC/NK crosstalk in the TME (264). These phenotypic and functional TME alterations due to MDSC/NK cell and/or T cell interactions contribute to pro-tumor, pro-angiogenic, and pre-metastatic activities in the tumor. Further studies are necessary to elucidate the mechanisms involved in MDSC/NK cell interactions to identify potential therapeutic candidates or pathways to limit NK cell MDSC-mediated suppression in the TME. MDSCs secrete high levels of soluble factors, such as ROS, inducible nitrogen-oxygen synthase (iNOS), nitrogen oxide (NO), peroxynitrate, and ARG1 (144), and show an elevated endoplasmic reticulum stress response (132, 265) (Figure 2).

NO is a ubiquitous, water-soluble, gaseous transmitter, which plays an essential role in various physiological conditions, inflammation, and cancer (266, 267). NO can play different roles in regulating immunity depending on the exact circumstances of its secretion. The autocrine production of NO by NK cells can improve NK cell function, but when MDSCs produce NO, it plays a vital role in mediating immunosuppression (266, 267). Co-cultures of autologous NK cells and MDSCs from patients with cancer showed that MDSCs suppress FcR-mediated function and signal transduction, leading to reduced responses to monoclonal antibody (mAb) therapies, and inhibit the secretion of IFN-γ and TNF-α by NK cells. Elimination of MDSCs or abrogation of NO production can improve responses to mAb immunotherapy (228).

MDSCs also support tumor progression by inducing tumor angiogenesis through the release of VEGF, basic FGF (bFGF), prokineticin 2 (Bv8), and some MMPs (169, 172, 268–270) (Figure 2). Some studies showed that MDSCs in the TME produce high levels of MMPs, including MMP2, MMP8, MMP9, MMP13, and MMP14 (167, 268, 271, 272). MDSCs in the presence of high levels of MMP9 can promote VEGF function by raising its bioavailability (273). VEGF stimulates MDSCs via STAT3 in the TME and potentiates their immunosuppressive activity by expanding other immune cell populations (213, 274, 275) and stimulating the secretion of numerous additional angiogenic factors and chemokines, which further enhance MDSCs accumulation within tumors. IL-1β, C-C motif chemokine ligand 2 (CCL2), CXCL8, CXCL2, angiopoietin 1 and 2 (AGPTs), and GM-CSF (Figure 2) have been shown to contribute to MDSC-mediated angiogenesis and involve STAT3 for their expression (80, 276–278).

The major components of the TME are the endothelial cells of the blood and lymphatic vessels, fibroblasts, immune cells, and the ECM (2). During tumor development and progression, cancer and stromal cells often have restricted access to nutrients and oxygen. Most solid tumors have hypoxic regions due to abnormal vascularization and inadequate blood supply (279). The changes to cancer and stromal cells that are necessary for tumor progression in a hypoxic environment are attributed to HIF-dependent signaling. The HIF family of transcription factors includes HIF-1, HIF-2, and HIF-3. Signaling by HIF-1 and HIF-2 induces the expression of multiple pro-angiogenic factors (VEGF, angiopoietin-2 [ANG-2], phosphatidylinositol-glycan biosynthesis class F protein [PlGF], bFGF, and semaphorin 4D), and angiogenesis was promoted in MDSCs by HIF-1 through VEGF and S100 calcium-binding protein A8 (S100A8) (280).

The TME has high levels of adenosine during hypoxia and inflammation. Adenosine/adenosine receptor interactions increase immunosuppression and angiogenesis through immune cells (281, 282). MDSCs express increased levels of CD39 and CD73 under hypoxic conditions or TGF-β stimulation (283, 284). These enzymes can convert adenosine triphosphate (ATP) and adenosine monophosphate (AMP) to adenosine, resulting in increased adenosine levels in tumor lesions (Figure 2). Adenosine accumulation impairs IL-2 and Ly49D activation, NKp46-receptor crosslinking, and maturation in NK cells (285). It has also been shown that adenosine signaling is involved in reducing the engagement of A2A adenosine receptor (A2AR) as a checkpoint in NK cell maturation (286).

Tregs interact with different components of the TME (287) and exert their suppressive function via contact-dependent and -independent mechanisms, which have been previously reviewed (288, 289). NK cell–Treg crosstalk in the human tumor has not been studied extensively (290). However, it has been shown that peripheral Tregs isolated from healthy donors and patients with gastrointestinal stromal tumors impaired NK cells by downregulating the expression of NKG2D activated receptors and also inhibited NK cell functions via membrane-bound TGF-β (291) (Figure 2). A similar effect was observed *in vitro* with cervical carcinomas (292).

Through these mechanisms, MDSCs and Tregs inhibit CTL and NK cell activity, promote tumor progression, and hinder antitumor immunity (169, 172, 287, 293–296). Therefore, it is not surprising that high MDSC infiltration of tumors correlates with poor patient prognosis and resistance to immunotherapy and chemotherapy (150, 175, 297–300). MDSC and Treg numbers also positively correlate with disease stage and tumor burden (114, 132, 144, 163–166, 201–203) and are predictors of poor outcome in patients with solid tumors (301–304). Both MDSCs and Tregs increase suppressive activity via signaling pathways, and their interactions in tumors have recently been reviewed (287, 305). Consistent with these findings, pharmacological targeting of MDSCs and Tregs in animal models and cancer patients significantly improves antitumor immunity, enabling tumor control (306, 307).

## Targeting MDSCs and Their Ligands—Crosstalk With NK Cells

The complexity of the TME impairs immune cell functions and affects their phenotype. Several strategies have been developed, investigated, and applied in clinical trials to target MDSC immunosuppression and enhance NK cells' cytotoxic activity in the TME.

**Figure 3:**
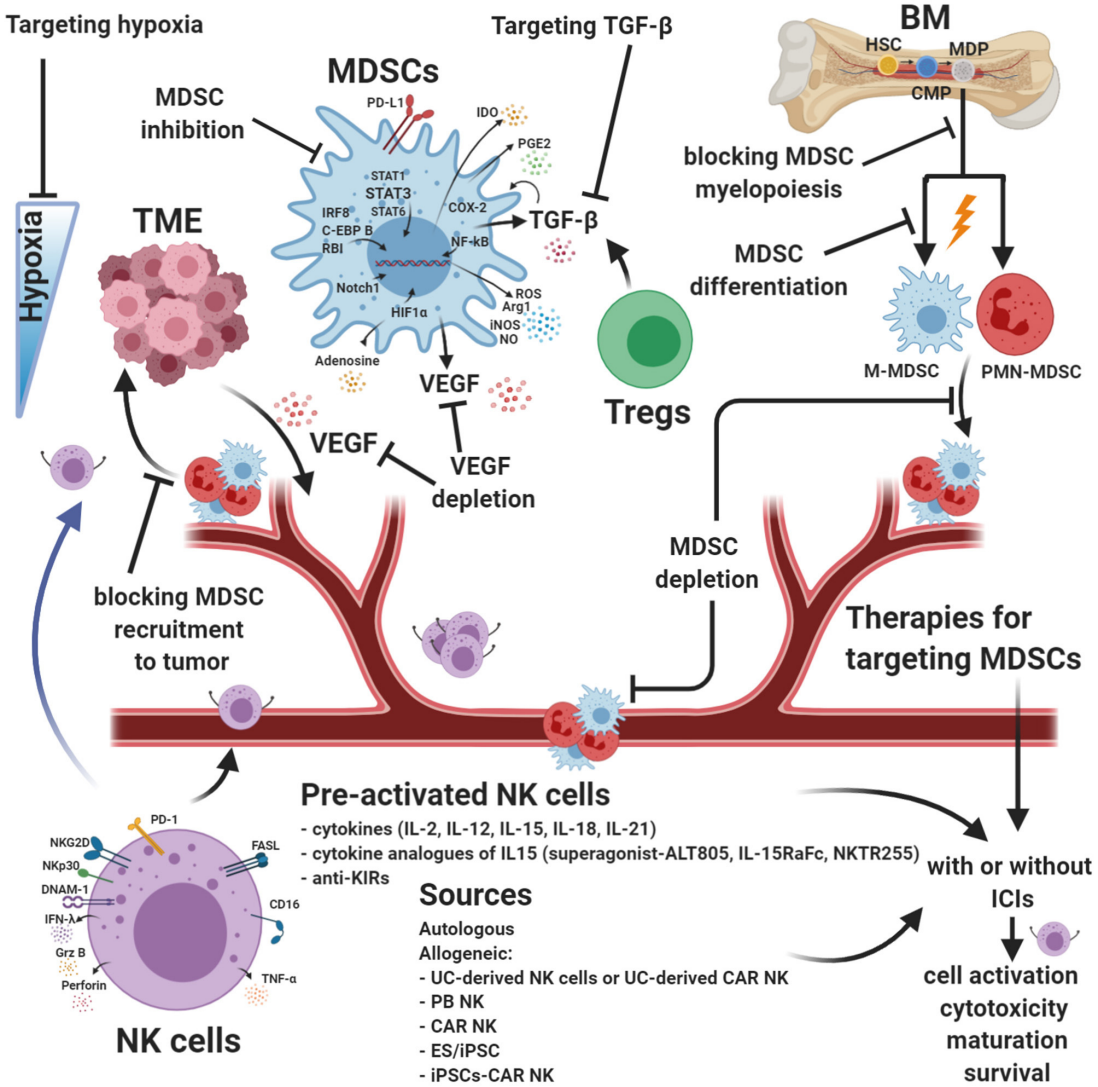
Strategies for targeting MDSCs and augmenting NK cell functions. Myeloid-derived suppressor cells (MDSCs) are robust immunosuppressors in the tumor microenvironment (TME) and represent an obstacle for many cancer immune therapies. Targeting MDSCs is a different approach designed to limit their immunosuppression within the TME and reduce neo-angiogenesis. Many combinatorial therapies are focused on blocking intratumoral recruitment and expansion of MDSCs. Other approaches are dedicated to increasing MDSC differentiation or inhibiting their immunosuppression activity. Natural killer (NK) cell-based immunotherapy strategies have been studied in an increasing number of clinical trials. Although NK cells can be useful in some types of cancer, it is necessary to improve the efficacy of currently available NK cell products to avoid cell exhaustion. Innovative combinatorial approaches intended to improve NK cell function and block MDSCs are in development. MDSC targeting, enhanced NK cell cytotoxic activity, and drugs targeting hypoxia can be used with current cancer therapies, including immunotherapy, and may improve antitumor response efficacy. ICIs, immune checkpoint molecules. Created with BioRender.com.

Studies have indicated that the types of mediators responsible for the differentiation, inhibition, and recruitment of MDSCs into the tumor were dependent on the different MDSCs subsets and tumor models (175). Targeting MDSCs is an approach designed to limit their immunosuppression within the TME and reduce neo-angiogenesis. Many therapies are focused on blocking intratumoral recruitment and expansion of MDSCs (Figure 3). Other approaches are dedicated to increasing MDSC differentiation or inhibiting their immunosuppression activity (264) (Figure 3). MDSCs are robust immunosuppressors in the TME and are an impediment for many cancer immune therapies (161).

Low doses of chemotherapy induce MDSC exhaustion (308, 309). Chemotherapy drugs such as gemcitabine (310), 5-Fluorouracil (311), and doxorubicin (312) reduce MDSC frequency, which enhances NK-mediated antitumor cytotoxicity (313–315). Phenformin and metformin, two antidiabetic drugs, impair MDSC functions mainly by blocking 5' AMP-activated protein kinase (AMPK). These drugs also upregulate the expression of MHC class I polypeptide-related sequence A (MICA) and heat shock protein 70 (HSP70) on cancer cells through the phosphatidylinositide 3-kinase/protein kinase B pathway leading to NK cell activation (316). In addition to chemotherapy and radiotherapy, many studies have combined MDSC targeting methods with immune-based therapies to increase the antitumor effects (317). Encouraging progress has been made combining MDSC targeting with immunotherapy strategies.

Blocking TGF-β pathways is a promising strategy in some preclinical and clinical trials. Some of the approaches currently under investigation in preclinical models and clinical trials consist of decreasing circulating TGF-β, blocking ligand–receptor interactions using neutralizing antibodies, and inhibiting TGF-β signaling pathways (NCT00356460 and NCT01722825) (318–323). Blocking TGF-β is a viable strategy to prevent myeloid precursors from differentiating into M-MDSCs and impair the migration and expansion of MDSCs and Tregs in the TME (Figure 3). Alternatively, manipulating NK cells to block TGF-β signaling pathways is an attractive and promising strategy for solid tumors. In SMAD3-silenced NK cells (NK-92-S3KD), TGFβ1-mediated immunosuppression was blocked, inhibiting cancer progression in mouse models with human hepatoma and melanoma (324). Similarly, NK-92 cells genetically modified to express a chimeric TGF-β type II receptor (extracellular and transmembrane domains) and the intracellular domain of NK cell-activating receptor NKG2D were resistant to TGF-β-induced suppressive signaling and did not downmodulate expression of NKG2D (325). The same authors demonstrated that NK-92-TN cells inhibited the differentiation of human naïve CD4^+^ T cells to Tregs and decreased tumor volumes *in vivo* in a hepatocellular carcinoma xenograft cancer model (325).

A recent study demonstrated that NK cell sensitivity to TGF-β can be reduced by stimulation of NK cells with IL-2 (Figure 3), which increases IFN-γ and TNF-α production by NK cells in the tumor, compared to NK cells that encountered acute TGF-β exposure or were not TGF-β imprinted (326). A new approach to overcoming tumor resistance mechanisms to ICIs has been tested by combining TGF-β, CXCR1/2 signaling, and PD-L1 (327). This simultaneous inhibition reduced mesenchymal tumor features and infiltration of suppressive PMN-MDSCs into the TME, improving antitumor activity by promoting immune cell infiltration and activation in tumors (327).

Cytotoxic agents and tyrosine kinase inhibitors (TKIs) deplete MDSCs and regulate myelopoiesis (264). TKIs, such as axitinib, sorafenib, and sunitinib, induce DNA damage by histone γ-H2AX phosphorylation and checkpoint kinase 1 activation, leading to senescence of human renal carcinoma cells (328). The presence of DNA damage in cancer cells also improved the identification of tumor cells by NK cells (328). TKIs can directly target VEGF and/or involve c-KIT signaling and interact with other factors, such as CSF or STAT3. These mechanisms impaired MDSC function and inhibited tumor angiogenesis (329, 330). These processes upregulate NKG2DLs and, consequently, stimulate NK cell antitumor cytotoxicity (329, 330). Several *in vivo* studies combined TKI and ICI therapies; the combination therapy improved antitumor mechanisms and simultaneously reduced immune cell exhaustion (330–333).

STATs are activated in tumor cells by multiple soluble factors. This activation causes impaired cytolytic functions mediated by perforin and granzyme B in NK cells and alters the expression of NK cell receptors NKG2D and DNAX accessory molecule-1 (DNAM-1) (232). STAT3 activation in tumor cells also represses the expression of NK cell-chemotactic factors, which reduce the migration of NK cells in the TME. TGF-β and IDO produced by tumor cells and MDSCs impair NK cell development, proliferation, and activation (232), leading to reduced NK-mediated cytotoxicity. Exhausted NK cells and T cells have a diminished secretion of IFN-γ (334). MDSCs secrete type I interferons to maintain a high level of PD-L1 expression and preserve their immunosuppressive activity in the TME as a compensatory mechanism (335); therefore, the autocrine IFNα/IFNβ-pSTAT1-PD-L1 circuit represents a pivotal pathway to targeted MDSCs (335).

STAT3 also increases the expression of PD-L1, which engages PD-1 expressed on NK cells, reducing their antitumor response (232). Janus kinase (JAK)/STAT3 inhibitors decrease MDSC trafficking in the TME, diminishing angiogenesis by inhibiting VEGFA and casein kinase 2 (336) and driving NK cell activation (336, 337). Targeting STAT3 in tumor-bearing mice leads to tumor reduction, better survival, and a significantly higher number of activated NK cells following treatment, as compared to control mice (252, 253).

Other approaches using STAT3 inhibitors combined with ICIs, such as nivolumab (NCT03647839), STAT3 small interfering RNA (siRNA), or decoy STAT3 oligonucleotide inhibitors alone or combined with ICIs (AZD9190), are in phase I/II clinical trials (NCT03421353). STAT3 siRNAs coupled to CpG oligonucleotides reduced the immunosuppression of toll-like receptor 9 (TLR9)-expressing PMN-MDSCs in preclinical data (338). Mouse and human PMN-MDSCs overexpress fatty acid transporter protein 2 (FATP2) due to stimulation by GM-CSF through activation of the STAT5 transcription factor (339). FATP2 inhibitors alone, or in combination with ICIs, delayed tumor progression in tumor-bearing mice (339). CSF-1R inhibitors, combined with anti-PD-1, improved the immune response in a mouse model of neuroblastoma (340).

MDSC immune functions are also impaired using class I deacetylase, entinostat, or ARG1 small-molecule inhibitors by decreasing iNOS and COX-2 levels (341). Some of these treatments enhanced NK killing and blocked MDSC-mediated suppression of T cells *in vitro* and *in vivo* in tumor models (342–344). One study showed that an ARG1 small peptide inhibitor combined with anti-PD-L1 slowed tumor growth (345). ARG1 inhibitors (such as CB-1158) showed encouraging preclinical results (346), increasing tumor-infiltrating NK cells and CD8^+^ T cells (345, 347), reducing tumor burden (348, 349), and decreasing MDSCs recruitment into the TME (345).

Conventionally, phosphodiesterase-5 (PDE5) inhibitors (sildenafil and tadalafil) are used as therapies for non-malignant conditions (350). Recent evidence suggests that PDE5 inhibitors could improve antitumor cell responses by inhibiting the suppressive functions of MDSCs in the TME (306, 351, 352). Preclinical and clinical data show that the PDE5 inhibitor tadalafil enhanced the immune response in head and neck squamous cell carcinoma (HNSCC) patients through inhibition of MDSCs (NCT01697800) (352). However, another PDE5 inhibitor, Sildenafil, reduced PMN-MDSC function through downregulation of ARG1, IL4Ra, and ROS expression (248). PDE5 inhibitors also enabled NK cell antitumor cytotoxicity and reduced postoperative disease recurrence. These studies have also shown Treg reduction and enhanced CD4/CD8 T cell function in the TME (248, 306, 352). Phase I/II Clinical Trial NCT02544880 used tadalafil as a therapy for decreasing MDSCs and Tregs, improving the antitumor response. The treatment might also enhance antitumor mucin 1 (MUC1) vaccine efficacy in patients with resectable and recurrent HNSCC by promoting a permissive environment (NCT02544880).

All-trans retinoic acid (ATRA) and vitamin A metabolites (retinol) have been used to treat acute myelogenous leukemia (353–355). Several studies reported that ATRA inhibited cell migration, metastasis, and proliferation, and promoted apoptosis of tumor cells (356–358). ATRA alone (359–361) or in combination with a DC vaccine against p53 (360) or IL-2 administration (361) showed lower MDSC frequencies and enhanced differentiation of MDSCs (359–361) into mature DCs, macrophages, and neutrophils (362). ATRA decreased the expression of immunosuppressive genes through the downregulation of TGF-β, PD-L1, IL-10, and IDO in MDSCs and upregulated MHC class I homologs MICA and MICB on tumor cells, enhancing NK cell activity (363, 364) and cytotoxicity of T cells (365) in the CD8^−^ and CD4^−^ immune response (362). In clinical studies, ATRA alone or combined with ipilimumab significantly reduced the level of circulating MDSCs in advanced-stage melanoma patients (366). In a preclinical breast cancer model, ATRA therapy improved the efficacy of anti-angiogenic treatments (367).

VEGF is produced by tumor cells in the TME and supports neo-angiogenesis, metastasis dissemination, and also acts as a chemoattractant for MDSCs in the tumor site (302, 368, 369). This has been shown in both NSCLC and renal cell carcinoma patients, especially under hypoxic conditions (370, 371). In a NSCLC model, VEGF attracts MDSCs from the BM to the periphery, expanding their presence throughout the individual (372). The binding of VEGF to its receptor is correlated with increased production ROS via the JAK2/STAT3 activation pathway. MDSCs can also secrete VEGF (Figure 2), creating, together with the tumor cells, a positive autocrine feedback loop in the TME (373, 374). Therefore, anti-VEGFR2 reduced the accumulation of intratumoral MDSCs, decreased hypoxia, and interfered with the formation of tumor microvessels through S100A8 (367). Genetic inactivation of VEGFA in MDSCs improved clearance of senescent tumor cells by NK cells, inhibited tumor regrowth after chemotherapy and, prevented cachexia in tumor-bearing mice (375). A phase II clinical trial evaluated the efficacy and pharmacokinetics of bevacizumab, an anti-VEGF recombinant human mAb, combined with capecitabine and paclitaxel chemotherapy in subjects with triple-negative, metastatic, or locally advanced breast cancer. In 77% of patients, the therapy showed an objective response rate, with complete response in 19%, and the median progression-free survival was 7.6 months (376). Several studies also demonstrated that MDSCs possess secondary pro-angiogenic mechanisms involving MMPs, as discussed in Vetsika et al.' review (162).

Because MDSCs are recruited to the TME by tumor cells, blocking their migration using a CCR5 antagonist and CCL2 inhibitors seems to be a promising therapeutic approach. Accumulation of CCR5^+^ MDSCs with high suppressive activity, associated with increased concentrations of CCR5 ligands and tumor progression, has been shown in a tumor-bearing melanoma study model (377). The upregulation of CCR5^+^ on CD11b^+^Gr1^+^ myeloid cells was induced *in vitro* by CCR5 ligands and other inflammatory factors. Blocking the CCR5/CCR5 ligand interaction improved survival by reducing the migration and the immunosuppressive functions of MDSCs in melanoma lesions of tumor-bearing mice (377). This strategy can also enhance the suppression of MDSCs by NK cells, as shown in a premetastatic lung animal model (378). CCL2 expression in MDSCs has been elucidated in a lung tumor model, and anti-CCL2 treatment can decrease peripheral and intratumoral PMN-MDSCs and M-MDSCs by inhibiting the ARG1 expression and iNOS (379). The same study showed enhancement in CD4^+^ and CD8^+^ T cell infiltration and production of IFN-γ in the TME. As a result, anti-CCL2 therapy increased the survival time of tumor-bearing mice. Anti-CCL2 therapy could be a potential approach to improve NK cell activity and the efficacy of ICI immunotherapy.

Some studies have shown that COX-2 inhibitors (celecoxib or nimesulide) reduced the expansion of MDSC subtypes and decreased cancer progression (380–382). COX-2 inhibitors also stimulate NKG2D ligand expression on tumor cells, enhancing NK cell-mediated cytotoxicity (383) and reducing angiogenic pathways via VEGF (384). A phase II clinical trial showed that perioperative treatment with a COX-2 inhibitor (etodolac) in combination with a β-adrenergic antagonist (propranolol) reduced circulating CD14^+^ monocytes and improved NK cell activation (385). Thus, this study supports the rationale for targeting MDSCs in the perioperative period to enhance clinical outcomes. A decrease in MDSC COX-2 and PEG2 activity and NO and ROS production has been shown in animals and humans after Vitamin D3 or E treatments (386). MDSCs impair NK cell function through NO production, and clinical trials are evaluating novel therapies to block this mechanism (228). A clinical trial using celecoxib, in combination with nivolumab and ipilimumab, is currently recruiting for the treatment of colon carcinoma (NCT03026140). A colorectal cancer phase III trial combining vitamin D3 with standard chemotherapy and bevacizumab is also ongoing (NCT04094688). Vitamin E supplementation decreased PGE2 production by inhibiting COX-2 activity, resulting from decreased NO production, in mice and humans (386). It also improved the activation of T and NK cells, increased lymphocyte proliferation, and modulated DC function (386).

ROS molecules are involved in many pathways, thereby controlling a wide range of biological events, such as immunosuppression in the TME (387). Following pathogenic and inflammatory immune responses, MDSCs release ROS (387). Although ROS has toxic effects on most cells, MDSCs survive despite the elevated levels and constant production of ROS in the TME (388). The production of ROS by MDSCs is upregulated in many murine tumor models and human cancers (389, 390) and plays a crucial role in maintaining MDSCs in an undifferentiated state (387). ROS production is regulated by the nuclear factor (erythroid-derived 2)-like 2 (Nrf2). Synthetic triterpenoid C-28 methyl ester of 2-cyano-3,12-dioxooleana-1,9,-dien-28-oic acid (CDDO-Me; bardoxolone methyl) in an MC38 colon carcinoma model has been shown to reduce MDSC ROS production via Nrf2 activation (307) and IL-17D production (391). A phase I clinical trial showed encouraging results using this therapy (392). Forcing IL-17D production using Nrf2 agonists can enhance NK cell activation and recruitment, leading to tumor regression (391, 393).

The pathological intratumoral accumulation of CXCR2^+^ PMN-MDSCs impairs the functions of NK cells in mice bearing oral carcinoma tumors by secreting TGF-β, NO, and ROS (394). CXCR1/2 inhibitors (SX-682) significantly abrogated MDSCs trafficking within a tumor and improved tumor infiltration, activation, and therapeutic efficacy of adoptively transferred murine NK cells in the HNSCC preclinical model (394), and in combination with anti-PD-1, they improved ICI therapy (395).

HIF-1α, released in the TME from tumor cells, is another protein that has a crucial role in MDSC differentiation toward TAMs (178, 396). Some progress has been achieved targeting hypoxia using hypoxia-activated prodrugs (TH-302) (Figure 3), hypoxia-modulator drugs (modulating expression, DNA-binding, transcriptional activity, and degradation of HIF proteins), drugs directly modulating HIF mRNA (antisense oligonucleotides), and inhibiting pathways involved in the control of HIF-1α mRNA (397). Combination therapy of TH-302 with gemcitabine in a phase II clinical trial for advanced or metastatic pancreatic cancer (NCT01144455) (398) or with doxorubicin in a phase III trial for advanced soft tissue sarcoma (NCT01440088) (399) showed encouraging results. The combination of TH-302 therapy with anti-CTLA-4 and PD-1 cured more than 80% of tumors in a mouse prostate cancer model by reducing MDSCs and granulocytic subsets and driving T cell migration into the hypoxic tumor sites (400). Innovative combinatorial approaches, such as drugs targeting hypoxia used with current cancer therapies, including immunotherapy and MDSCs targeting, may improve antitumor response efficacy.

IL-18 and IL-33 are involved in CD11b^−^ BM progenitor cell differentiation to M-MDSCs (192) and PMN-MDSCs (401), respectively. Targeting of CD11b^−^ BM treated with anti-IL-18 or IL-33 has been shown to enhance T cell proliferation and IFN-γ secretion (192, 401). Other MDSC molecules targeting aminoacyl tRNA synthetase complex interacting multifunctional protein 1, TLR agonists, tumor-derived exosome-associated HSP72, and inflammasome component NLR family pyrin domain containing 3 have also been shown to contribute to MDSC differentiation and have been reviewed previously (402). MDSCs can be blocked by combining ICIs (anti-PD-1, PD-L1, and CTLA-4) with anti-IL-18 (192), inhibitors of FATP2 (339), long non-coding RNA Pvt1 (403), downregulation of the pseudogene Olfr29-ps1 (404), or deletion of the nuclear factor 1 A gene (405).

## Pre-Activated NK Cell Products for Cancer Immunotherapy

Cancer immunotherapy strategies have focused on T-cell-based immunotherapy using expanded tumor-specific CD8^+^ CTLs from tumor-infiltrating lymphocytes (TILs) (406). Adoptive transfer of TILs following lymph-depleting strategies have shown promising effectiveness in metastatic melanoma studies (407–409), but short-lived responses (410) and side effects, such as vitiligo, uveitis, and retinitis, have been reported (407, 411).

Pre-clinically and clinically, studies have identified several cytokines and other novel soluble factors that increase NK cell numbers, function, and persistence. The two most common strategies are pretreating NK cells with cytokines (before the adoptive transfer) or *in vivo* cytokine administration. IL-2 (412), IL-15 (413), IL-12 (414, 415), IL-18 (416, 417), IL-21, their combinations, and administrations with other immunotherapeutic agents have all been described in the context of regulating NK cell function, maturation, survival, and improving activation and cytotoxicity (Figure 3). Results are expected from clinical trials evaluating the safety and efficacy of combining IL-21 with other immunotherapeutic mediators: IL-21/anti-PD-1 against solid tumors (NCT01629758) and IL-21/ipilimumab against melanoma (NCT01489059). Cytokine analogs of IL-15, such as IL-15 superagonist ALT-803 (NCT03228667, NCT03127098, NCT03022825, NCT02384954, NCT02138734, NCT02890758, NCT02559674, and NCT03520686) and NKTR-255, are under investigation. Furthermore, multiple ongoing clinical trials are evaluating the safety and efficacy of several other immune cytokines alone or in combination with other therapeutic strategies, such as immune checkpoint inhibitors (NCT03209869, NCT03386721, NCT02627274, and NCT02350673) (Figure 3).

Combination therapies of PD-1 blockade and IL-15 stimulation and also IL-15 and IL-15RaFc have been reported as safe in mouse models (418) and patients with NSCLC (419). IL-15 stimulation increases the expression of the activating receptors CD16 and NKG2D on NK cells and increases the activation, proliferation, cytotoxic activity, and survival of NK cells and CTLs (420). One of our preclinical studies found that the combination of PD-1 blockade and IL-15 signaling resulted in eradication of transplanted lung adenocarcinoma (LUAD) cells in about one-half of treated LUAD-SIS-PDX mice, while the other half presented with a partial response. Notably, IL-15 alone, without PD-1 blockade, significantly reduced tumor burden in all treated LUAD-SIS-PDX animals (418). PD-1 blockade alone transiently prevented tumor growth, but tumors grew at a similar rate to untreated control tumors after 2 weeks. The addition of IL-15 to PD-1 blockade completely abrogated tumor escape from ICI, resulting in a powerful additive therapeutic effect capable of tumor eradication. These findings support a key role for adjuvant IL-15 treatment to induce an immune cell-mediated tumor attack, which can prevent tumor escape from checkpoint blockade therapy, as shown using our novel LUAD-SIS-PDX model (418).

In addition to cytokine stimulation, mAb can be used to block the activity of NK cell inhibitory receptors. The ligation of inhibitory KIRs by HLA molecules triggers NK cell inhibition (64), and anti-KIR antibodies are under investigation to improve NK cell cytotoxicity in cancer (421). Because the expression of NK cell-expressed inhibitory KIRs and PD-1 correlate in patients with solid tumors (e.g., NSCLC), combining anti-KIR antibodies with anti-PD-1 treatments to avoid immune escape of tumors in these patients may be an effective treatment strategy (422). Several clinical trials are currently evaluating anti-KIR antibodies against solid tumors combined with other immune treatments (NCT03341936, NCT03203876, and NCT03347123).

## NK Cell Sources

NK cells do not need prior activation to target tumor cells. The fine-tuning of NK cell functions occurs during their maturation and instills a form of tolerance. NK cells are “educated” to recognize healthy MHC-I-expressing cells with KIR receptors, resulting in inhibitory signals and preventing NK cell activation. In addition to KIRs, NK cells also express two other types of inhibitory receptors (423): leukocyte immunoglobulin-like receptors (LILRs) (424) and C-type lectin receptors (NKG2A/CD94). The balance of inhibitory and activating signals expressed on target cells mediate NK cell activation and response (425–427).

NK cells can kill tumor target cells through a variety of mechanisms, as discussed previously in this review. Since NK cells represent an important defense against tumors, NK cell infusion products have been evaluated as a possible cancer immunotherapy. Different NK cell sources have been tested in patients with tumors combined with chemotherapy (428) and, allogeneic NK cells have been selected in many studies for their increased alloreactivity, achieved by mismatching of inhibitory KIRs and tumor HLA (429) (Figure 3). This is referred to as a haploidentical or half-matched setting. Haploidentical NK cells are more reactive against recipient tumor cells because of reduced KIR-mediated inhibition (429). NK cells for adoptive transfers can be obtained from five different sources: autologous NK cells, allogeneic NK cells (430), umbilical cord-derived NK cells, NK cell lines, and embryonic stem cell-derived/induced pluripotent stem cell (ES/iPSC)-derived NK cells (431) (Figure 3), allowing for a multitude of choices to match or mismatch KIR-HLA receptor pairings.

## Chimeric Antigen Receptor–NK Cells for Cancer Immunotherapy

One currently promising strategy is redirecting NK cells with chimeric antigen receptors (CARs) (Figure 3). Autologous CAR-T cell successes in patients with leukemia and lymphoma (432) have raised considerable interest in using immune cells as a cancer treatment. The advantage of a CAR strategy is that one CAR can be applied for many tumor types expressing the matching ligands. Modification with CAR is also proposed for reprogramming NK cells to improve their cytotoxicity. CAR-NK cells represent an exciting approach for cancer immunotherapy. NK cells can be targeted with CARs against surface molecules expressed by tumor cells and might avoid some of limitations or side effects of CAR-T cells. While conventional T cells are HLA-restricted, CAR-T cells are designed to recognize their target antigens independent of HLA expression and deliver their costimulatory signal. However, CAR-T cells are expensive and labor-intensive to generate (433), and the application of CAR-T cell therapy is often limited by intrinsic risks, such as graft-vs.-host disease (GvHD) (434). Also, off-target effects, cytokine release syndrome, and other side effects restrict their clinical applications. CAR-T cell therapy has been successful in treating blood cancers. However, a significant obstacle for the treatment of solid tumors is an extremely immunosuppressive TME that decreases the ability of immune cells to infiltrate tumors (435). CAR-T cell functions are often suppressed in solid tumors due to T cell expression of PD-1 and PD-L1 expression in the TME. These data may explain why the use of CAR-T cells has not been as useful for treating solid tumors as it has been to treat hematological malignancies (436). However, PD-1 levels expressed by NK cells are substantially lower, making NK cells good candidates for eradicating solid tumors (437).

Allogeneic NK cell transplantation rarely induces GvHD (438) and has the potential to become “off-the-shelf” products, making CAR-NK cell therapies a possible widespread product (433). Large-scale culture and genetic modification of allogenic human NK cells are feasible and could readily be used to treat a broad range of cancer patients. NK cell-expressed CARs typically include a single-chain variable fragment from a mAb, a transmembrane hinge region, and a costimulatory signaling domain, such as CD28, CD3-zeta, 4-1BB (CD137), or 2B4 (CD244) heterodimers (439, 440). These main signaling domains frequently have been derived from TCR moieties (441). Four generations of CARs are currently under development (442). The first generation of CARs usually contains only the CD3ζ activation signaling domain (443). In the second and the third generations of CARs, costimulatory molecules like CD28, 4-1BB, and CD134 are also included to increase NK cell activation (444). The fourth generation of CARs is engineered to secrete transgenic cytokine-like IL-12, which should help to remodel the tumor environment to promote therapeutic success (442, 445). Once CAR-modified NK cells recognize their specific targets, such as CD19^−^, CD20^−^, or CD138^−^ cells, the CAR receptors trigger an intracellular signaling cascade that activates CAR-NK cells to kill the antigen-expressing target cell.

Methods to generate CAR-NK cells that target solid tumors include lentiviral transduction or electroporation of the NK-92 NK-like cell line (438), primary NK cells, and the differentiation of NK cells from modified pluripotent stem cells (431). NK cells express NKG2D, an activating receptor triggered by MICA/B and UL binding protein (ULBPs) expressed on the surface of stressed cells upon DNA damage, hypoxia, or viral infection (446). NKG2D ligands are often overexpressed on solid tumors and tumor-infiltrating cells like MDSCs (447). However, the NKG2D cytotoxic adapter molecule, DNAX-activation protein 10 (DAP10), is downregulated by suppressive molecules like TGF-β, which is abundantly expressed in the TME (448). NK cells and CAR-NK cells expressing the native NKG2D receptors are thus downmodulated in the TME due to the reduction in DAP10 expression. To overcome the repressive effects of the solid TME on NKG2D functions, one group (449) established a gene-modified NK cell bearing a chimeric receptor in which the activating receptor NKG2D is fused to the cytotoxic ζ-chain of the T-cell receptor (NKG2D.ζ) (450). This specific CAR has been designed to target MDSCs in the TME of solid tumors, which are refractory to other types of immunotherapy. The NKG2D.ζ-NK cells are cytotoxic against MDSCs, but unmodified NK cells are not. They also showed that NKG2D.ζ-NK cells generated from patients with neuroblastoma successfully killed autologous MDSCs in the TME, which were capable of suppressing CAR-T functions (449). CAR-NK cells have been established and engineered against several antigens for solid tumors, including epidermal growth factor receptor, human epidermal growth factor receptor-2 (HER2), egeria, disialoganglioside, epithelial cell adhesion molecule, mesothelin, and tyrosine-protein kinase transmembrane receptor ROR1, with promising results in preclinical or clinical studies (39). In a recently published phase I/II clinical trial, HLA-mismatched anti-CD19 CAR-NK cells were administered to 11 patients with relapsed or refractory CD19-positive cancers. The majority of treated patients showed a response to treatment with anti-CD19 CAR-NK cells without developing significant toxic side effects (451). The phase I/II trial was approved for B-cell lymphoma in 2017 (NCT03056339). In another study, NK cells derived from umbilical cord blood were transfected with a CAR containing inducible caspase 9/IL-15 (iC9/CAR.19/IL-15) (452). They produce IL-15, which supports CAR-NK cell survival, and are engineered to express the inducible suicide gene caspase 9 for their pharmacologically induced elimination. Liu and coworkers showed efficient iC9/CAR.19/IL-15 cell killing of CD19-expressing tumor cell lines *in vitro* and improved clinical outcome in a xenograft Raji lymphoma murine model (452). In the clinical trial, iC9/CAR.19/IL-15 cells were used to treat patients with relapsed/refractory CD19^+^ B lymphoid malignancies. This cell therapy was applied with high-dose chemotherapy (NCT03579927).

An alternative approach for the generation of CAR-NK cells is the use of iPSC as a platform to generate CAR-NK cells (453) (Figure 3). In 2018, a NK cell line was established from human iPSCs that expresses a CAR containing the transmembrane domain of NKG2D, the 2B4 costimulatory domain, and the CD3ζ signaling domain able to mediate strong antigen-specific NK cell signaling (iPSC-CAR-NK cells) (453). The combination of NKG2D-2B4ζ in this CAR construct conferred strong upregulation and activation of phospholipase C gamma, Syk-vav1-Erk, and NF-κB pathways, improving iPSC-CAR-NK cell activation, proliferation, and antitumor activity. This cell line maintains the same NK cell phenotype as wild-type cells but enhanced antitumor activity compared to CAR-T cells, iPSC-NK cells, or non-CAR PB-NK cells. In an ovarian cancer xenograft model, iPSC-CAR-NK cells significantly inhibited tumor growth and improved survival in mice and showed less toxicity than CAR-T cells (453).

A pilot study investigated the use of NKG2D ligand targeted CAR-NK cells in patients with metastatic colorectal cancer and evaluated the safety and feasibility of CAR-NK cell treatment against solid metastatic tumors (454). For these studies, autologous or allogeneic NK cells were transfected by mRNA electroporation to generate CAR-NK cells with transiently enhanced specificity and activity against NKG2D ligand-expressing cancer cells. This approach has also been used in a clinical trial involving solid metastatic tumors (NCT03415100).

About 200 clinical trials are currently using CAR-T cells, and only 20 clinical trials so far are utilizing CAR-NK cells (http://www.clinicaltrials.gov). For these trials, CAR-NK cell products are derived from either primary NK cells or NK cell lines. Only five clinical trials were conducted to evaluate the safety of CAR-NK-92 infusion products, while 15 trials evaluated CAR-NK cells from other sources. CD7 (NCT02742727), CD19 (NCT02892695), and CD33 (NCT02944162) targeted blood cancer (455); HER2 targeted glioblastoma (NCT03383978); co-stimulating conversion receptors targeted NSCLC (NCT03656705); and MUC1 targeted multiple refractory solid tumors, including hepatocellular carcinoma, NSCLC, pancreatic tumors, and triple-negative metastatic breast tumors (NCT02839954) (455).

Additional preclinical or clinical trials targeting CD19^−^ (NCT03690310) or CD22^−^ expressing (NCT03692767) cells are ongoing, and bivalent CD19/22 (NCT03824964) CAR-NK cell products are also being examined in patients with relapsed and refractory B cell lymphoma (456). One clinical trial targeting CD19-positive cells is testing CD19-CAR-NK cell infusions in a pediatric setting (NCT00995137), using irradiated K562-mb15-41BBL expanded PB-derived NK cells transfected with CD19-41BBz-CAR (455).

Roundabout homolog 1 (ROBO1), a member of the axon guidance receptor family (Robo1–4), is a potential target for immunotherapy (457). ROBO1 modulates the chemotaxis of T cells and tumor angiogenesis to counteract the tumor growth (458–460). Some solid tumors, such as pancreatic cancer, have increased expression of ROBO1 (461). Three clinical trials are currently studying CAR-NK cells directed against ROBO1, using CAR-NK (NCT03940820), bi-chimeric antigen receptor-NK cell BiCAR-NK (NCT03941457), and bi-chimeric antigen receptor-NK cell or T cell BiCAR-NK/T (NCT03931720) cells on patients with solid tumors (456).

Additional strategies for optimizing NK cell-mediated cytotoxicity have been employed. ADCC is a potent mechanism of cytotoxicity used by NK cells. An increasing number of mAbs are currently being examined in preclinical and clinical studies for their ability to improve antitumor ADCC (462). Limiting shedding of CD16 from NK cell membranes also enhances ADCC, and iPSC-NK cells modified to overexpress a non-cleavable version of CD16 showed improved ADCC *in vitro* and *in vivo* in a human B-cell lymphoma model (463). iPSC can also be modified to improve *in vivo* persistence of iPSC-NK cells. Furthermore, the deletion of cytokine-inducible SH2-containing protein (CISH) in iPSC differentiated NK cells improved persistence and enhanced antitumor activity in a leukemia xenograft model (464). Other relevant strategies to strengthen NK killing potential use antibody therapy to target NK cell checkpoints that inhibit NK cell activity in the TME, such as PD-1 (465), T cell immunoreceptor with Ig and ITIM domains (466), and single Ig IL-1-related receptor (467). These findings provide some flexibility in the combining of therapeutic approaches. NK cells can be modified to express CARs and/or be used in combination with ICI or additional antibody immunotherapy. While significantly more work is needed to optimize these approaches, these data nevertheless demonstrate the potential for “off-the-shelf” NK cell platforms for treating solid tumors and hematological malignancies. Further research needs to be done to identify and understand possible CAR-NK exhaustion mechanisms after transplantation in preclinical and clinical studies.

## Conclusion

The TME plays a critical role in regulating NK cell antitumor functions and regulating NK cell trafficking, persistence, proliferation, and activation. MDSCs are one of the critical players inducing and regulating the immunosuppressive environment of the TME. MDSCs interact with numerous innate immune cells, modulating their functions and suppressing strong tumor-specific immunity. As such, MDSCs represent an essential target in oncology. While NK cell-MDSC interactions have been investigated, few studies have evaluated how NK cell cytotoxicity can be exploited to attack both the immunosuppressive TME as well as the tumor. We have provided an updated review of several current prospective therapies for targeting the MDSC–Treg axis and improving antitumor NK cell functions (several mechanisms are summarized in Figure 3). These pathways have substantial implications for the tumor and are currently under investigation. The combinatorial treatments that target these pathways have been showing promising results. More work is needed to fully exploit NK cell functions to eradicate hematological malignancies and solid tumors.

## Author Contributions

CZ and SP conceptualized the content and wrote the article. All authors contributed to the article and approved the submitted version.

## Conflict of Interest

The authors declare that the research was conducted in the absence of any commercial or financial relationships that could be construed as a potential conflict of interest.
